# Secondary Metabolites in Durian Seeds: Oligomeric Proanthocyanidins

**DOI:** 10.3390/molecules181114172

**Published:** 2013-11-15

**Authors:** Yuancai Liu, Shengbao Feng, Lixia Song, Guangyuan He, Mingjie Chen, Dejian Huang

**Affiliations:** 1 Hubei Key Laboratory of TCM Based Functional Food Quality and Safety, Jing Brand Company, Daye 435100, China; E-Mail: lyc@jingpai.com; 2 College of Life Science and Technology, Huazhong University of Science and Technology, Wuhan 430074, China; E-Mails: hegy@mail.hust.edu.cn (G.H.); cmj@mail.hust.edu.cn (M.C.); 3 National University of Singapore (Suzhou) Research Institute, Suzhou Industrial Park, Suzhou 215123, China; E-Mails: lisasong82@gmail.com (L.S.); chmhdj@nus.edu.sg (D.H.); 4 Food Science and Technology Program, Department of Chemistry, National University of Singapore, 3 Science Dr. 3, Singapore 117543, Singapore

**Keywords:** durian seeds, oligomeric proanthocyanidins, thiolysis, polyphenolic compounds, MALDI-TOF MS

## Abstract

Ethanolic extract of durian seeds was fractionated by reverse phase flash column chromatography and the fractions characterized by electrospray ionization mass spectroscopy. Among a few unknown compounds collected, oligomeric proanthocyanidins (OPCs) were found to be one of the main compounds. Based on this result, the OPCs were purified the first time from the durian seeds using standard procedures and gave a yield of 1.8 mg/g dry matter after fractionation by Sephadex LH-20 column. Structural analysis by ^13^C{^1^H} NMR and ESI-MS spectra showed the presence of primarily B-type procyanidins with mainly epicatechin as the extension units, which was further verified by matrix assisted laser desorption/ionization–time of flight mass spectra (MALDI-TOF MS), which shows a distribution of dimers to decamers. In addition, hydroxylated peaks with molecular weight 16 units more than the poly-epicatechins represented significant peaks. We suggest this might be due to hydroxylation occurring under the MALDI-TOF MS conditions. Consistently, depolymerization with α-toluenethiol resulted in epicatechin thioether as the major product, but undetectable amount of gallocatechin or its α-toluenethiol derivatives. The oligomershave a mean degree of polymerization of 7.30.

## 1. Introduction

Flavonoids are a prominent group of polyphenolic compounds that has been studied intensively [1]. Among the various subclasses of flavonoids, proanthocyanidins (condensed tannins, OPCs) are important component of many fruits and plant leaves [2,3]. There has been an increase in interest in OPC research in recent years owning to their wide spectrum of pharmacological action [4], such as their excellent antioxidant activity, free radical scavenging activity, inhibition of platelet aggregation, anti-ulcer activity, and radioprotective effects [4,5,6,7].

**Figure 1:**
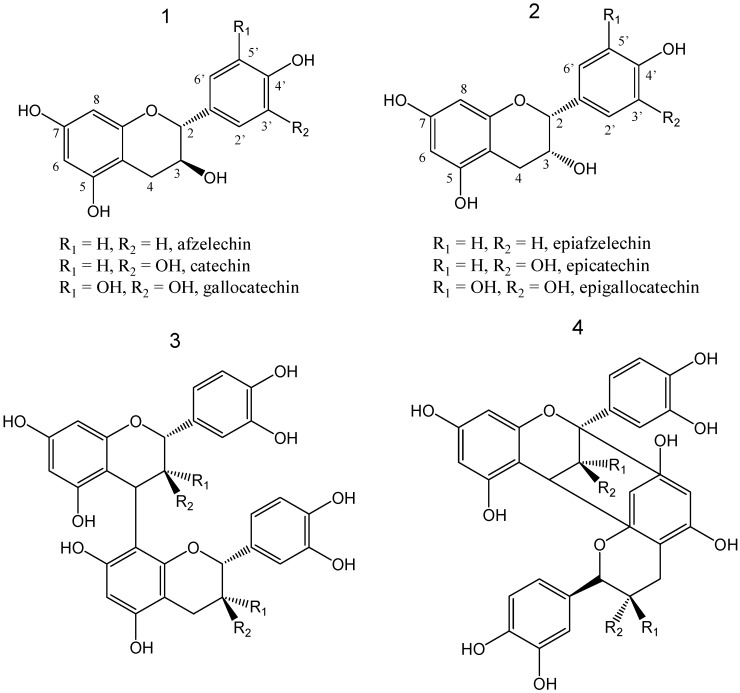
Structures of the flavan-3-ol building blocks (**1** and **2**) of proanthocyanidins, B-type (**3**) and A-type (**4**) dimeric blocks of proanthocyanidins. B-type (dimeric) are characterized by single linked flavanyl units between C-4 and C-8 (3) or C-6 (not shown), while A-type possess an additional ether linkage between C-2 of the upper unit and a 7- and/or 5-OH of the lower unit. Where R_1_ = H and R_2_ = OH, catechin; R_1_ = OH and R_2_ = H, epicatechin [3,6].

Proanthocyanidins are oligomers and polymers of the flavan-3-ol monomer unit that are most commonly built up with repeat units of (epi)catechin, (epi)gallocatechin, or (epi)afzelechin (Figure 1) [3,6]. Bioactivities of proanthocyanidins are influenced by the diversity of their structures including the degree of polymerization, the content of epicatechin unit in the polymers, the stereochemistry and hydroxylation pattern of the flavan-3-ol starter and extension units, and the position of the linkage between two monomeric units (A type OPCs or B type OPCs, Figure 1) [5,7].

Durian (*Durio zibethinus* Murr.) is a seasonal fruit grown in Southeast Asia that belongs to the Bombacaceae family [8]. Widely known in Southeast Asia as the “King of Fruits”, durian is distinctive for its large size, unique odor and taste, and formidable thorn-covered husk. Only one-third of the durian fruit is edible, whereas the seeds and the shell are discarded as agricultural wastes. Most research work hass focused on the flavor, phenolic contents and other nutritional properties of the edible portion of durian [9,10,11,12,13], and very rare research was concerned with the systematic characterization of the bioactive secondary metabolites in durian seeds, although some reports mentioned its use by local people to treat sores and wounds [14], or the collection of durian seed gum and fatty acids as agricultural waste [15,16,17]. Frequent climate changing-caused agricultural crisis have highlighted the importance of utilizing renewable agricultural wastes as sources of value-added food ingredients and bioactive compounds. This prompted our study on the bioactive secondary metabolites of durian seeds. Our research found that durian seed contains a specific content of oligomeric proanthocyanidins, which may be a prospective source of novel bioactive compounds. Reported herein are our results on isolation and structural characterization of the oligomeric proanthocyanidins identified in durian seed.

## 2. Results and Discussion

### 2.1. Chromatographic Fractionation of Durian Seed Extracts

**Figure 2:**
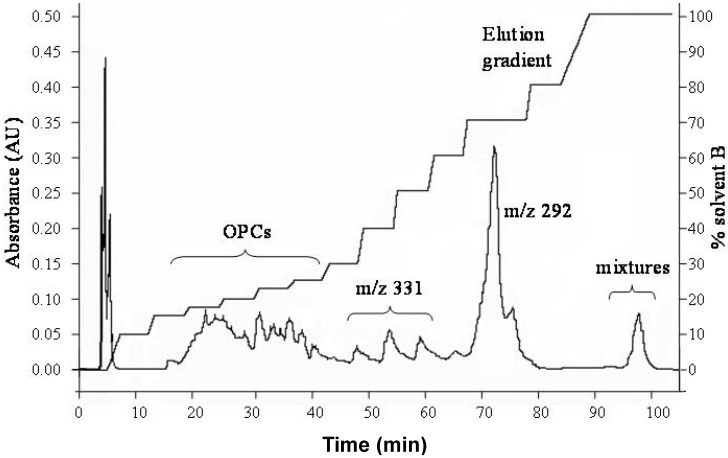
Flash chromatograms of durian seed extract (C-18 column) at a detection wavelength of 280 nm.

Nineteen fractions were collected by flash chromatography and a typical trace of a run is shown in Figure 2. The molecular weights of the peak clusters were identified by ESI-MS and are labeled in Figure 2. The clusters of peaks from 15 to 40 min all correspond to oligomeric proanthocyanidins. The three peaks from 45 to 60 min all shown major ESI-MS molecular ions at 331 (negative mode). A strong signal at 70 min gave a *m/z* 292 peak indicating it is a nitrogen-containing compound. Its exact chemical identity remains to be determined at this moment. A peak at 95 min is composed of mixtures according to the ESI-MS data. Therefore, oligomeric proanthocyanidins are the major components in the aqueous fraction of durian seed extract. Accordingly we carried out a systematic extraction and structural characterization.

### 2.2. Extraction and Structural Elucidation of OPCs

The durian seeds were extracted with acetone/water (7:3, v/v) to yield an optimal amount of OPCs. The crude proanthocyanidin fractions were purified by Sephadex LH-20 column chromatography to yield 362 mg of a brown mixture from 200 g freeze-dried durian seeds. The OPCs content in durian seeds is rather low as compared to that in mangosteen pericarp (6.63 mg/g dry weight), cocoa and grape seeds, which contain significantly higher levels of OPCs mixture of at least 100 mg/g dry matter.

**Figure 3:**
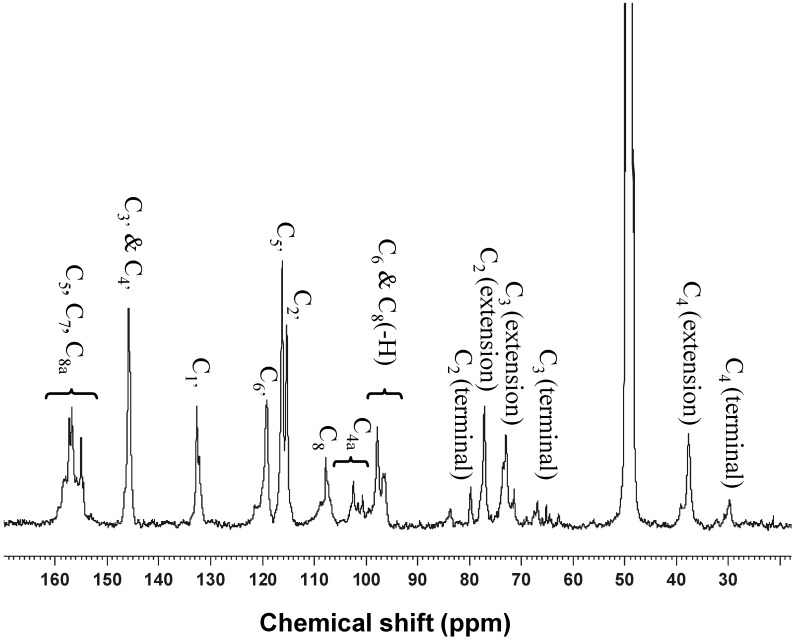
^13^C{^1^H}-NMR spectrum of proanthocyanidins from durian seed. Sample was dissolved in deuterate methanol and the data were collected at room temperature with operating frequency of 75 MHz.

The ^13^C{^1^H}-NMR spectrum of the extract in Figure 3 shows characteristic ^13^C peaks consistent with thaose of condensed tannins with dominant polymeric (epi)catechins with little impurities. Specifically, peaks clustered between 160–155 ppm were assigned to the C5, C7, and C8a carbons on the A rings. The B-ring carbons fall nicely in a narrow range between 150–110 ppm with distinctive sets of signals for C3' and C4' at 145 ppm, C1' at 132.2 ppm, and C6', C5' and C2' in the neighborhood of 120 ppm. All of them are fairly sharp peaks, indicating rather uniform poly-(epi)catechin structural motif. The C8 of the extension unit shows a peak at 107.5 ppm, whereas the C8 of the terminal unit gives a sharp line at 98 ppm close to that of C6. The few small lines between 100 and 105 ppm are due to C4a. The three aliphatic carbons in the C ring account for the rest of peaks starting with the terminal C2 at 80.0 ppm. The C2 of extension unit gives as sharp line at 77 ppm and C3 of the extension units are found at 73 ppm. The C3 of terminal unit gives rise to a small but sharp line at 66 ppm. Lastly, the C4 of extension unit is found at 37.5 ppm and that of terminal unit is located at 30 ppm. With the majority of peaks assigned to procyanidins, there is no detectable peaks were found for prodelphinidin or gallated procyanidins, indicating low concentration of these derivatives. 

**Figure 4:**
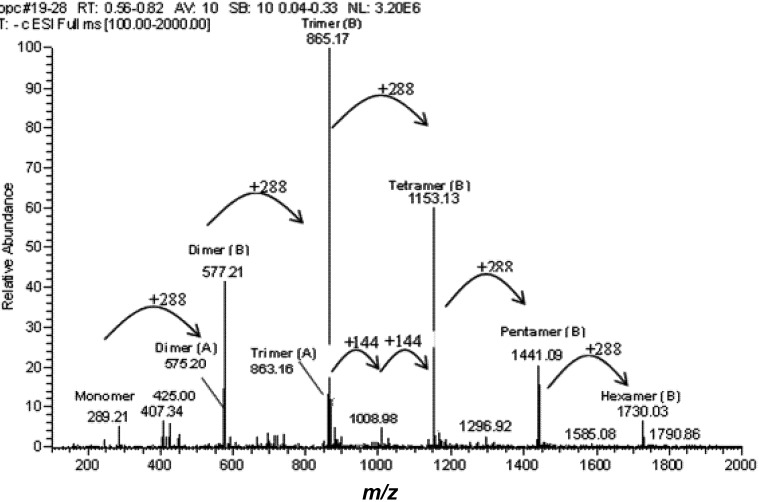
ESI-MS spectrum of purified OPCs collected under anionic mode. A series of peaks were detected with *m/z* differing 288 starting from monomer (289) and ending at hexamer (1,730). A type dimer and trimer were also detected as small peaks at *m/z* 575 and 863. A doubly charged haptomer was also found at 1,008.

In support of the ^13^C-NMR spectrum data, the ESI-MS data shown in Figure 4 provide further evidence of the durian seed OPCs lacking gallo or gallate units but being composed mainly of procyanidins, which give rise to a series of anions from *m/z* 289 to 1,730 separated by 288 Da. These sets of signals correspond to the molecular masses of procyanidins with degrees of polymerization (DPs) of 1–6. The mass spectra also shows a series of compounds at *m/z* 1,008, 1,296, and 1,585 that are 144 mass units higher than those described above. Es-Safi and coworkers reported that these signals can be explained by the presence of double charged ions [M−2H]^2−^ of odd polymerization degree, starting from DP 7 [18], but with lower abundance compared with the single charged ions. Such multiply charged species are reported to be more frequently observed in ESI-MS [19] and became more intense as the molecular weight increases, probably due to the longer chain length that allows a better charge separation to minimize the electrostatic repulsive forces. Signals corresponding to trimeric (*m/z* 882) and pentameric (*m/z* 1,169) oligomers with only one afzelechin/epiafzelechin unit (−16 Da) were also observed at low abundance. The mass spectra also show weak peaks for a group of compounds that were two units less than dimer (*m/z* 577) and trimer (*m/z* 865). These masses might represent a series of compounds in which an A type interflavan ether linkage occurs (4β-8, 2β-O-7) between adjacent flavan-3-ol subunits because two hydrogen atoms (Δ2 amu) are lost in the formation of this interflavan bond. Finally, a peak signal at *m/z* 425 can be accounted for by a fragment of procyanidin dimer (*m/z* 577) produced by a retro Dials-Alder fragmentation splitting the C ring. Another minor ion was detected at *m/z* 695 that did not match any of the known usual proanthocyanidins. The fact that it is 288 Da apart from *m/z* 407 suggested the existence of flavanol units in these compounds.

**Figure 5:**
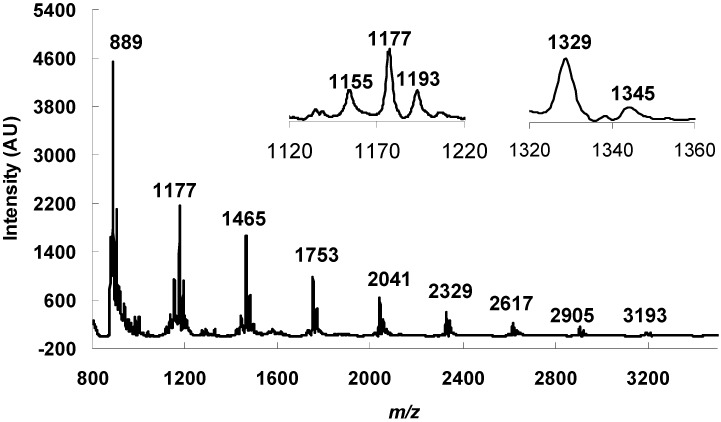
MALDI-TOF mass spectrum in positive linear mode, showing a procyanidin series [M+Na]^+^ from the trimer (*m/z* 889) to the nonamer (*m/z* 3,193). Inset is an enlarged spectrum of masses representing a procyanidin series with the presence of protonated trimer at *m/z* 1,155 and hydroxylated trimer at *m/z* 1,193. Small amount of gallated trimer were also detected at *m/z* 1,329 (=1177 + 152 (gallate)), and its hydroxylated compound at *m/z* 1,345.

MALDI-TOF MS was used as a complementary spectroscopic technique to identify the compounds with large molecular weight. This technique curbs the production of multiple charged species and enables elucidation of the polymer structures and chain lengths. Only single charged molecular ions are generated for each parent molecule, thus a better and more precise evaluation can be made. Figure 5 displays the MALDI-TOF mass spectrum of the OPCs mixture that shows a series of polyflavan-3-ols extending with distances of 288 Da from the dimer (889 *m/z*) to the 11-mer (*m/z* 3,193), which corresponded to the mass difference of one catechin/epicatechin extension unit between each polymer. Hence, the propagation of proanthocyanidins was due to the addition of catechin/epicatechin monomera. The absolute masses corresponding to each peak also suggested that they contain only procyanidins, as was already indicated in the ^13^C-NMR spectrum. The masses were calculated based on the following equation [20]:

*m/z* = 288n + 23 + a

where 288 corresponds to the molecular weight of one catechin unit, 23 is the molecular weight of sodium added. a is the number of ^1^H in the end groups (corresponds to 2). The number of monomer units is given by n.

Some minor signals warrant some attention. The trace amounts of gallated tetramers gave rise to a peak at *m/z* 1,329 (1,177 + 152) along with the corresponding hydroxylated compound at *m/z* 1,345. This is not detected in the ^13^C-NMR spectrum or ESI-MS as the concentration might have been too low. It is also remarkable that a few smaller peaks are observed next to each major peak of a given oligomer. The representative one at *m/z* 1,177 was shown in the inset of Figure 5. The peak (1,155) with 22 units less is a protonated tetramer. The intensities of the similar peaks at higher mass drop progressively as the degree of polymerization increases. The peak at 1,193 is a tetramer containing one more hydroxyl group, indicating the presence of one gallocatechin unit. Similar peaks are consistently present for all oligomers. In the anionic mode of ESI-MS spectrum, we only observe a very minor peak at *m/z* 1,169 for the hydroxylated tetramer. To probe the significance of gallocatechin units in the OPCs, we further examined the thiolyzed products by HPLC and LC-MS but again failed to detect significant amount of gallocatechin. Therefore, we suspect that the hydroxylation occurred under the MALDI-TOF MS experimental conditions. We suggest that OPCs or the 2,5-dihydrobenzoic acid may react with oxygen to give hydrogen peroxide. In the MALDI-TOF experiment, the samples were loaded in a stainless steel plate which may provide ferrous iron for a Fenton reaction that leads to hydroxylation of the OPCs. The laser bombardment on the sample may further facilitate such reactions. However, due to the small sample sizes used in MALDI-TOF MS measurements we were not able to verify this hypothesis.

### 2.3. Determination of the Degree of Polymerization Procyanidins by Thiolysis

**Figure 6:**
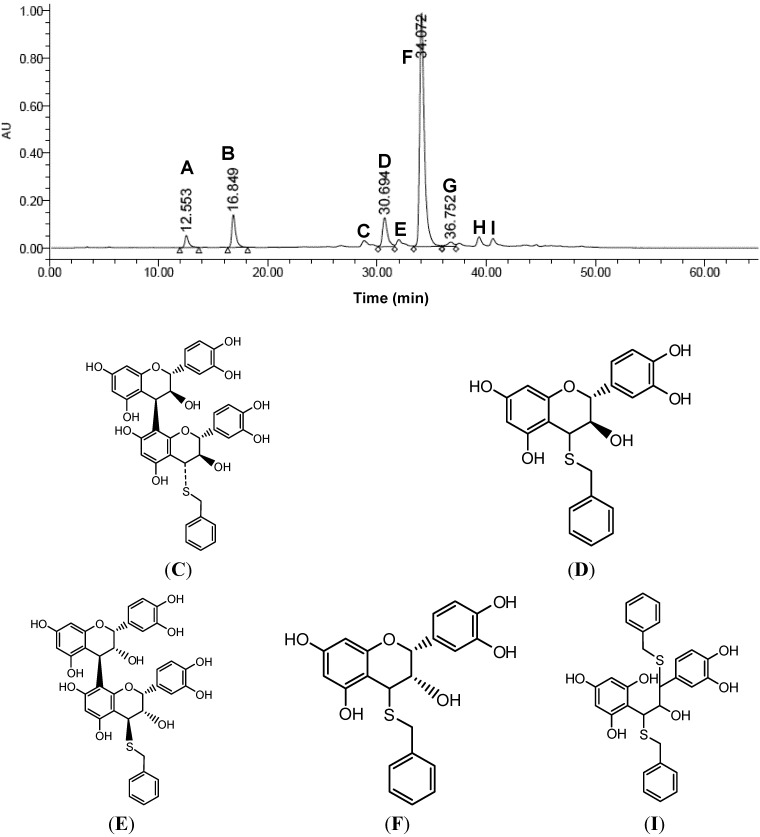
HPLC chromatogram (detector wavelength = 280 nm) of thiolytic products of durian seed OPCs by α-toluenethiol and the possible thiolytic products detected by LC-MS spectra. A, catechin; B, epicatechin; Structures of C, D, E, F, and I are shown in the Figure G is excess α-toluenethiol, H is an unknown compound.

The catechin and epicatechin composition of OPCs can be determined by the depolymerization of OPCs through thiolysis reactions (22; 18; 23). The addition of a thiolytic reagent, α-toluenethiol, results in an attack on the procyanidins’ extension subunits to form the corresponding benzylthioethers. Only the terminal unit is released as the free flavan-3-ol. Figure 6 shows the HPLC chromatogram after thiolysis. 

A large portion of the product observed was epicatechin 4-benzylsulfide alongside small peaks of epicatechin and catechin. Other thiolytic products were also detected in smaller peaks. The identity of the possible thiolytic products were further confirmed by LC/MS spectra (data not shown) to be catechin, epicatechin, thiolated dimer (C and E stereoisomers), thiolated catechin (D) and thiolated epicatechin (F, most dominant peak), and small amount of dithiolated catehin (I). However, no gallocatechin or gallated catechin is detected.

**Figure 7:**
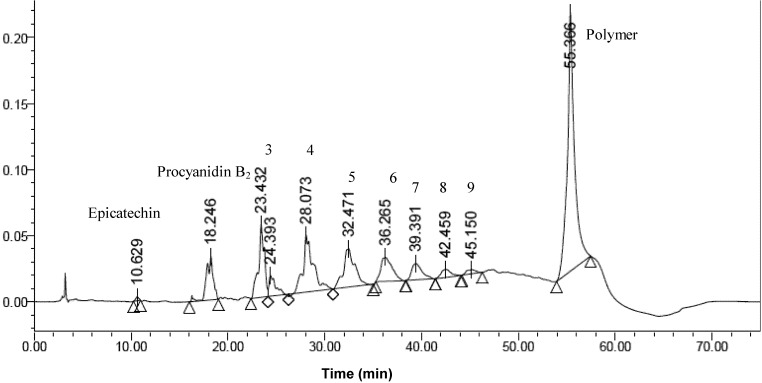
Normal phase HPLC separation of proanthocyanidins in durian seeds. The numbers above the peaks indicate the degree of polymerization of B-type procyanidins. The broad peaks are likely caused by the rotamers of proanthocyanidins arising from the hindered rotations of interflavanol C-C bonds.

The HPLC spectrum of the OPC thiolytic mixture shows a high concentration of epicatechin, indicating that this is the major monomeric unit. Using epicatechin as reference standard, the purity of the sample was calculated to be over 98%. The mean DP of the durian seed is calculated to be 7.3 based on the peak areas ratio of the thiolated epicatechin and the sum of catechin and epicatechin peaks [21]. The determination of mean DP values does not reflect the heterogeneity of the procyanidin mixture. The distribution of different procyanidins in the durian seed were quantified from a normal phase HPLC analysis shown in Figure 7, which gave satisfactory separation of the individual oligomers up to nonamer.

All the polymers with DP values larger than 9 were likely to be eluted at the same time without separation at 55.3 min. Catechin, epicatechin and procyanidin B2 were also analyzed. The peak at 10.63 min corresponded to epicatechin. The peak at 18.24 min corresponded to procyanidin B2 dimer. From the peak area, the low DP procyanidins can be quantified using epicatechin as a standard and expressed as epicatechin equivalents (ECE, Table 1). Tetramer and pentamer proanthocyanidins with dominant B type linkages constitute the majority of the procyanidins contents with 0.180 mg ECE/g and 0.141 mg ECE/g respectively in durian seed. This result was consistent with the obtained MS (Figure 4) whereby the highest relative abundance of the OPCs corresponded to trimer, tetramer, pentamer and dimer. It is noted that the peaks are rather broad for each oligomer. The rotamers arising from the hindered rotation of interflavanol bonds are likely to be the main contributing factor to this [22].

**Table 1:** Oligomeric proanthocyanidin profiles in durian seeds (unit: mg/g dried seeds).

Peak	Retention Time (min)	Concentration of procyanidins (μg/mL)	Content of procyanidins (mg ECE/g dry matter)
Monomers	10.63	1.06	0.000958
Dimers	18.25	86.24	0.0780
Trimers	23.43	137.74	0.125
Tetramers	28.07	199.39	0.180
Pentamers	32.47	155.39	0.141
Hexamers	36.27	104.56	0.0946
Heptamers	39.39	57.72	0.0522
Octamers	42.46	31.97	0.0289
Nonamers	45.15	4.96	0.00449
Polymers	55.37	763.38	0.690
Total			1.395

In summary, durian seeds contain complex secondary metabolites including OPCs that are dominantly B-type linked polymers of epicatechin ranging from dimer to at least 11-mers. Therefore, homogeneous OPCs may be a good source for preparing epicatechin derivatives through depolymerization reaction with nucleophiles such as thiols and carbon nucleophiles. The epicatechin derivatives have shown enhanced bioactivity in comparison with epicatechin itself. Besides OPCs, we also found that durian seeds also contain some novel secondary metabolites with molecular weight of less than five hundred whose structural characterization is warranted.

## 3. Experimental

### 3.1. Instruments

Separation of extract was done by flash chromatography using a CombiFlash^®^ Companion^TM^ (Teledyne ISCO, Linclon, NE, USA) automated purification system. High performance liquid chromatography (HPLC, analysis was carried out on a Waters HPLC system (Waters, Milford, MA, USA) with a 2996 PDA detector using a Shimadzu ODS-VP C18 column (4.6 × 250 mm, 5 μm particle size) (Shimadzu, Kyoto, Japan). LC/MS spectra were acquired using Finnigan/MAT LCQ ion trap mass spectrometer (Thermo Scientific, San Jose, CA, USA) equipped with a TSP 4000 HPLC system, which includes UV6000LP PDA detector, P4000 quaternary pump and AS3000 autosampler. The electrospray ionization mass spectra (ESI-MS) analysis was also conducted in the Finnigan/MAT LCQ ion trap mass spectrometer equipped with an ESI source. The heated capillary and spray voltage were maintained at 250 °C and 4.5 kV, respectively. Nitrogen is operated at 80 psi for sheath gas flow rate and 20 psi for auxiliary gas flow rate. The full scan mass spectra from *m/z* 50–2,000 were acquired both in positive and negative ion mode with a scan speed of one scan per second. Matrix-assisted laser desorption/ionization time-of-flight (MALDI-TOF) mass spectra were collected on an Applied Biosystems Voyager-DE STR mass spectrometer (Applied Biosystems, Foster City, CA, USA) equipped with delayed extraction and a N2 laser set at 337 nm. ^1^H- and ^13^C-NMR spectra were recorded with a Bruker AC300 spectrometer (Bruker Spectrospin GmBH, Karlsruhe, Germany) operating at 300 and 75 MHz, respectively.

### 3.2. Reagents

D24 cultivar durian seeds from Malaysia were purchased from a local durian puff shop and vacuum packed and stored at −30 °C freezer for further use. Ethanol, ethyl acetate, acetone, acetonitrile and methanol were purchased from Fisher Scientific (Pittsburgh, PA, USA) and Tedia Company Inc. (Fairfield, OH, USA). All the solvents used were of reagent grade unless further otherwise specified. Freshly prepared HPLC-grade mobile phases were sonicated for 15 min before HPLC analysis. Reference compounds including (+)-catechin, (−)-epicatechin and procyanidins B2 were purchased from Sigma Chemical Co. (St Louis, MO, USA).

### 3.3. Solvent Extraction and Fractionation of Durian Seeds

The durian seeds (500 g) were first blended in a blender, after which 50% ethanol was added to the slurry (5 mL/g). The extraction was continued by vigorous shaking on an orbital shaker for one hour and the mixture was then centrifuged at 8,000 rpm for 15 min. The residue was extracted again with 95% ethanol (1:1, v/v) and centrifuged at 8,000 rpm for 15 min. The supernatent of the two extracts were combined and concentrated to 1/10 of the original volume before acetone (1:1, v/v) was added to precipitate the proteins and gums. After removal of the precipitate by centrifuging at 8,000 rpm for 15 min, the supernatant was subjected to phase separation using ethyl acetate. Two fractions, the aqueous (more polar) portion and the EA (less polar) portion were collected separately. The aqueous extract was concentrated with rotary evaporator at 40 °C under reduced pressure and transferred to a reverse phase C18 column for crude separation in the CombiFlash^®^ Companion^TM^ automated purification system using a C18 reversed-phase (RP) column (RediSep^®^, ISCO, 4 gram, 230–400 mesh, average Pore Size: 100 angstroms, 1 cm × 8 cm.). A stepwise gradient comprises of water (A) and acetonitrile (B) as the mobile phase was used as follows: 0–5 min, 0% B; 5–7 min, 0%–15% B; 7–12 min, 15.0%; 12–13 min, 15%–20% B; 13–18 min, 20%; 18–19 min, 30%; 19–24 min, 30% B; 24–25 min, 30%–40% B; 25–30 min, 40%; 30–31 min, 40%–50% B; 31–36 min, 50%; 36–37 min, 50%–60% B; 37–42 min, 60%; 42–43 min, 60%–70% B; 43–48 min, 70%; 48–51 min, 70%–100% B; 51–70 min, 100%. The flow rate was 5 mL/min. UV-Vis monitoring was set at wavelength of 234 nm and 280 nm. During the separation, fractions (15 mL each) were collected and analyzed by reverse phase thin-layer chromatography (TLC C18 sheets 20 cm  ×  20 cm, F254, Merck, Darmstadt, Germany) with use of the same eluent and gradient as for the flash chromatography. Fractions containing the same compound were combined and solvents were evaporated. The residue was subject to ESI-MS characterization.

### 3.4. Extraction and Purification of Oligomeric Proanthocyanidins from Durian Seeds

After indentifying the present of proathocyanidins in durian seeds from the above extraction and fractionation method, we carried out a systematic extraction and structural characterization accordingly. Freeze-dried durian seed (200 g) was used for extraction using the published method for extraction of oligomeric proanthocyanidins from mangosteen pericarp [21]. The freeze-dried sample was defatted with hexane (600 mL) three consecutive times. The mixture was stirred on an orbital shaker for two hours each time. The slurry was then centrifuged at 8,000 rpm for 15 min. The solid residue was collected and subsequently extracted with 70% acetone (600 mL) three times. The mixture was filtered through a Millipore 0.45 μm PTFE filter membrane and the filtrate was pooled. Acetone was removed by rotary evaporation at 40 °C under reduced pressure. The residue was collected for further liquid-liquid extraction. The extraction was carried out with dichloromethane (600 mL) on the orbital shaker for two hours to remove lipophilic compounds. The extraction was repeated for three times. The aqueous phase was collected and concentrated to 40 mL. The crude proanthocyanidin fraction was loaded onto a Sephadex LH-20 column (2.5 × 20 cm), which contained 50 g of LH-20 and was equilibrated with 50% methanol for 4 h before sample load. The column was washed continuously with 50% methanol to remove sugar until the eluent turned colourless. Desorption of the proanthocyanidins were then eluted with aqueous acetone (70%, 500 mL). The acetone was removed using a rotary evaporator under reduced pressure at 40 °C, and the resultant residue was freeze-dried to give a dark-brown powder. Before subsequent analysis, the OPCs powder (2 mg) was further dried in the vacuum oven for 24 h (room temperature) and dissolved in deuterated methanol (1 mL) for NMR identification. The data were collected at room temperature with operating frequency of 75 MHz.

### 3.5. Oligomeric Proanthocyanidins Thiolysis and Identification

The thiolysis reaction was conducted in a small glass vial according to the reported method [23]. The durian proanthocyanidins solution (50 μL, 2.0 mg/mL in methanol) was mixed with acidified methanol (3.3% hydrochloric acid, 50 μL) and α-toluenethiol (5% v/v in methanol, 100 μL). The vial was sealed with an inert Teflon cap. The reaction was carried out at 40 °C for 30 min and then kept at room temperature for 10 h to ensure complete degradation. The reaction mixtures were kept in the freezer (−30 °C) to prevent further epimerization or other side reactions before HPLC analysis was carried out on a reverse phase Shimadzu ODS-VP C18 column (4.6 × 250 mm, 5 μm particle size). The binary mobile phase consisted of solvents A (2% acetic acid in water, v/v) and B (methanol). The flow rate was set at 1.0 mL/min. A linear gradient of 0–1min, A 85%; 1–50 min, A 85%–20%; 50–60 min, A 20%–85%; 60–65 min, A 85% was used. Standard (+)-catechin, (−)-epicatechin and procyanidins B2 were also analyzed using the same HPLC condition. LC-MS method was conducted to identify other portion of thiolyzed products using the same HPLC gradients.

### 3.6. Quantitative Analysis of OPCs using Normal Phase HPLC

The quantitative analysis was conducted using a Waters HPLC system with a 2996 PDA detector using Phenomenex Luna Silica column (4.60 × 250 mm, 5 μm particle size) column connected with a silica guard column according to the reported method [24]. The tertiary mobile phase comprises of (A) dichloromethane, (B) methanol, and (C) 50% acetic acid in water (v/v). Gradient was adapted from the reported method [24] with some modifications: 0–20 min, 14.0%–23.6% B; 20–50 min, 23.6%–35.0% B; 50–55 min, 35.0%–86.0% B; 55–60 min 86.0% B isocratic; 60–65 min, 86.0%–14.0% B, followed by 10 min of re-equilibration of the column before the next run. A constant 4.0% (C) was kept throughout the gradient. The detection wavelength was 280 nm.

## 4. Conclusions

Durian seeds contain complex secondary metabolites including OPCs that are dominantly B-type linked polymers of epicatechin ranging from dimer to at least 11-mers. Therefore, homogeneous OPCs may be a good source for preparing epicatechin derivatives, which have shown enhanced bioactivity in comparison with epicatechin itself. Durian seeds have the potential to be reused as a prospective value-added food ingredients and bioactive compounds instead of discarding as waste. 

## Abbreviations

PC: Procyanidins
OPCs: Oligomeric proanthocyanidins
ESI-MS: electron spray ionization-mass spectrometry
MALDI-TOF MS: matrix assisted laser desorption/ionization–time of flight mass spectrometry
ECE: epicatechin equivalent
